# Epitopes of an antibody that neutralizes a wide range of SARS-CoV-2 variants in a conserved subdomain 1 of the spike protein

**DOI:** 10.1128/jvi.00416-24

**Published:** 2024-04-16

**Authors:** Hanako Ishimaru, Mitsuhiro Nishimura, Hideki Shigematsu, Maria Istiqomah Marini, Natsumi Hasegawa, Rei Takamiya, Sachiyo Iwata, Yasuko Mori

**Affiliations:** 1 Division of Clinical Virology, Center for Infectious Diseases, Kobe University Graduate School of Medicine, Kobe, Hyogo, Japan; 2 Structural Biology Division, Japan Synchrotron Radiation Research Institute SPring-8, Sayo, Hyogo, Japan; 3 Division of Cardiovascular Medicine, Hyogo Prefectural Kakogawa Medical Center, Kakogawa, Hyogo, Japan; Loyola University Chicago - Health Sciences Campus, Maywood, Illinois, USA

**Keywords:** severe acute respiratory syndrome-coronavirus 2 (SARS-CoV-2), Omicron variants, human monoclonal antibody, broadly neutralizing activity, spike, subdomain 1, cryoelectron microscopy, common epitope, vaccine

## Abstract

The evolution of severe acute respiratory syndrome coronavirus 2 (SARS-CoV-2) has continued, enabling the virus to escape from host immunity by changing its spike antigen, while biased toward the receptor-binding domain and N-terminal domain. Here, we isolated a novel pan-SARS-CoV-2 neutralizing antibody (which we named MO11) for even the recent dominators XBB.1.16 and EG.5.1, from a convalescent patient who had received three doses of an original mRNA COVID-19 vaccination. A cryo-electron microscopy analysis of the spike-MO11 complex at 2.3 Å atomic resolution revealed that it recognizes a conserved epitope hidden behind a glycan shield at N331 on subdomain 1 (SD1), holding both the N- and C-terminal segments comprising SD1. Our identification of MO11 unveiled the functional importance of SD1 for the spike’s function, and we discuss the potential availability of a novel common epitope among the SARS-CoV-2 variants.

**IMPORTANCE:**

Novel severe acute respiratory syndrome coronavirus 2 variants with immune evasion ability are still repeatedly emerging, nonetheless, a part of immunity developed in responding to the antigen of earlier variants retains efficacy against recent variants irrespective of the numerous mutations. In exploration for the broadly effective antibodies, we identified a cross-neutralizing antibody, named MO11, from the B cells of the convalescent patient. MO11 targets a novel epitope in subdomain 1 (SD1) and was effective against all emerging variants including XBB.1.16 and EG.5.1. The neutralizing activity covering from D614G to EG.5.1 variants was explained by the conservation of the epitope, and it revealed the importance of the subdomain on regulating the function of the antigen for viral infection. Demonstrated identification of the neutralizing antibody that recognizes a conserved epitope implies basal contribution of such group of antibodies for prophylaxis against COVID-19.

## INTRODUCTION

**Fig 1:**
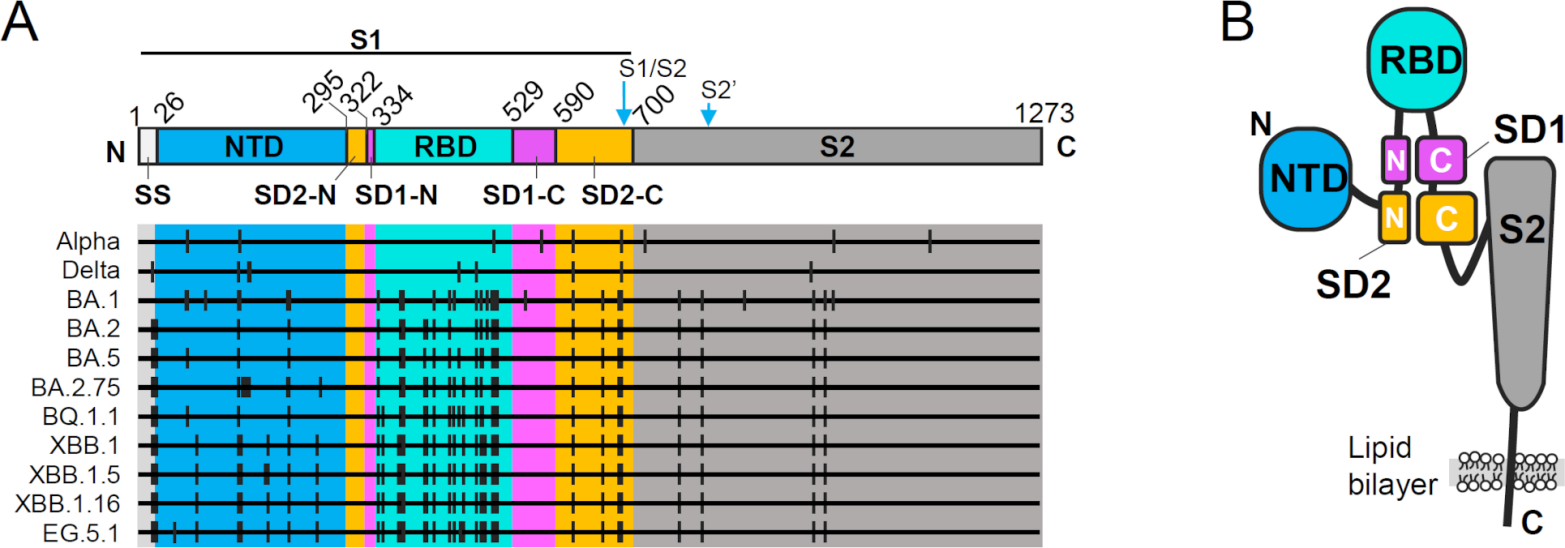
Domain structure of the severe acute respiratory syndrome coronavirus 2 (SARS-CoV-2) spike antigen and reported mutations. (**A**) Structural domains of the spike antigen considering each fold in the 3D structure and mutation sites in major SARS-CoV-2 variants. NTD, N-terminal domain; SD1, subdomain 1; SD2, subdomain 2; RBD, receptor binding domain. Mutation positions are shown for the selected major variants. The S1/S2 and the S2 cleavage sites are also indicated. (**B**) A schematic illustration of a spike protomer, showing the spatial arrangement of the domains. SD1 and SD2 were composed of two distant parts, unlike the other domains.

The spike antigen of severe acute respiratory syndrome coronavirus 2 (SARS-CoV-2) is a key molecule in the control of coronavirus disease 2019 (COVID-19). The continuous evolution of SARS-CoV-2 since the emergence of the virus at the end of 2019 has been driven by increasing mutations in the spike via interactions with host immunity, especially with neutralizing antibodies elicited by infection and/or vaccination. In the 1,273 amino acid residues of the spike, mutations are concentrated mostly in the receptor-binding domain (RBD), which binds to the host-receptor angiotensin-converting enzyme 2 (ACE2) (1), and numerous mutations have also been identified in the N-terminal domain (NTD) of the virus (Fig. 1A and B), especially after the emergence of the Omicron variant at the end of 2021. The biased mutation frequency indicated that neutralizing antibodies targeting these domains are imposing selection pressure in human bodies, but SARS-CoV-2 has escaped this pressure by undergoing ingenious modifications in the epitopes without losing their function.

The SARS-CoV-2 spike protein forms a trimer and serves as a molecular machinery that undergoes dynamic structural changes during infection on the SARS-CoV-2 virion. The spike has a furin cleavage site at RRAR_682-685_ and is divided into S1 and S2 domains during virion assembly and maturation while tethered by a single-pass transmembrane domain at the C-terminal region (Fig. 1A and B). The S1 domain contains the NTD and an RBD as well as small domains called subdomain (SD)1 and SD2, and the S2 domain forms a helical core of the trimer. The NTD and RBD each consist of a continuous region and form a single fold exposed at the tip of the spike, and SD1 and SD2 are separated in the primary structure (Fig. 1A and B). The pre-fusion spike trimer is not static, as the three RBDs take so-called “up” and “down” (or “open” and “closed”) conformations to expose or hide the receptor-binding motif in the RBD, respectively. The spike trimers normally all take “down” forms or a mixture of “up” and “down” conformations for the three RBDs in the trimeric fold as revealed by cryo-electron microscopy (cryoEM) (2, 3) and cryo-electron tomography (4). In light of the multifunctional and dynamic features of the spike (5, 6), it is possible that the neutralizing antibodies elicited by host immunity use a variety of neutralizing mechanisms in addition to the inhibition of receptor engagement.

Our previous attempt to isolate cross-neutralizing antibodies from human blood cells led to the identification of a prospective neutralizing antibody, MO1, that can neutralize the BA.5 variant by targeting a conserved epitope in the RBD (7). However, two newly emerged variants, XBB.1 and BQ.1.1, escaped from MO1 as well as other antibodies by harboring mutations in the RBD. Considering the fact that the SARS-CoV-2 keeps evolving by gaining mutations in the RBD and NTD (Fig. 1A), further explorations of target sites on the spike and additional knowledge about the inactivation mechanism of the spike molecule are required.

SD1 is an architectural keystone of the spike molecule (Fig. 1A and B), and it is almost free from mutation in the reported SARS-CoV-2 variants—even in the recent dominant variant, XBB.1.16 (Fig. 1A). In the trimeric form of the spike, the SD1 is in wide contact with the NTD and the S2 of the adjacent protomer, implying a role of maintaining the trimer’s structure. Since the SD1 tethers the RBD, the receptor engagement event will be transmitted to the other domains via the SD1, and a displacement of SD1 from the adjacent NTD and S2 is necessary for linkage with the dynamic conformational transition of the spike trimer as the “open trimer” state suggested by Costello et al. (5).

However, the potential of SD1 as a target for SARS-CoV-2 neutralizing antibodies has not been established as only limited reports are available to date (8–12), in contrast to the extensive studies of many neutralizing antibodies targeting the RBD as well as the NTD (13). Interestingly, most of the rarely reported SD1-targeting antibodies derived from a human source recognize their epitopes only when the spike trimer loosens the inter-protomer interaction, as the “open trimer.” The available epitope of SD1 is limited because of the originally small surface area, sequestration by S2 and the NTD of the adjacent protomer, and the glycan shield covering the exposed surface on the spike trimer (14, 15).

In the present study, we identified a monoclonal antibody (mAb) which we named MO11; it has broad neutralizing activity against SARS-CoV-2 variants including the recent variants XBB.1.16 and EG.5.1. The cryoEM structure of the MO11-spike trimer complex with C3 symmetry was determined at 2.3 Å global resolution, and we unveiled a fully conserved epitope behind a gatekeeping glycan shield on SD1. We discuss the implications linking the demonstrated neutralizing activity and the binding site of MO11 based on MO11’s binding mode on the spike trimer.

## RESULTS

### Human monoclonal antibody MO11 has neutralizing activity against a broad range of SARS-CoV-2 variants

**Fig 2:**
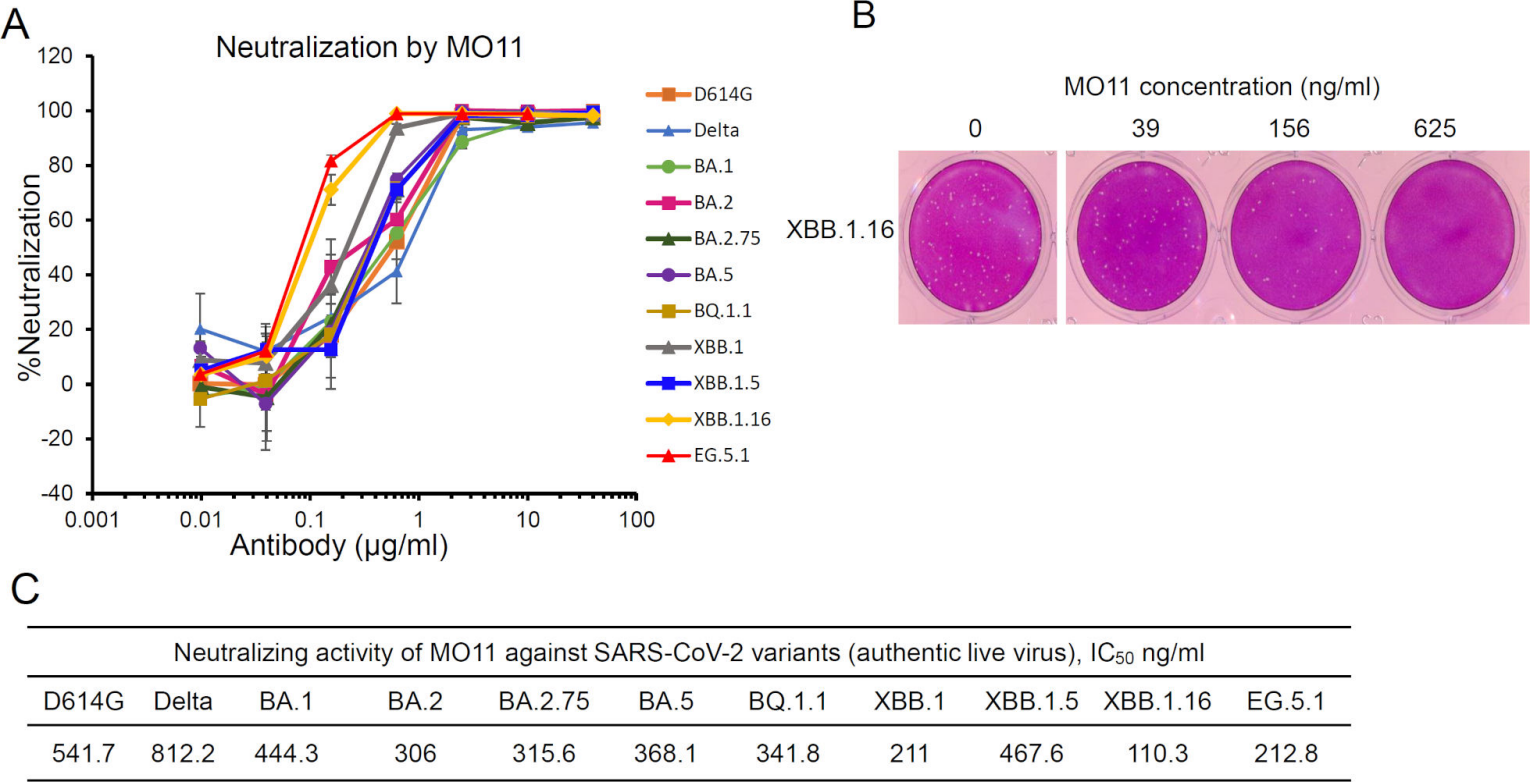
MO11 can neutralize a broad range of severe acute respiratory syndrome coronavirus 2 (SARS-CoV-2) variants including the recent variants XBB.1.16 and EG.5.1. (**A**) Plaque reduction assay for authentic SARS-CoV-2 variants. The neutralization rate for infection to VeroE6 TMPRSS2^+^ cells is plotted for the MO11 concentration. The mean values with standard errors are plotted. Each data point is representative of two or three independent experiments. (**B**) Representative pictures showing the plaque reduction by MO11. (**C**) Table of the IC_50_ values calculated from the plot in panel (**A**).

A human monoclonal antibody, MO11, was isolated from B cells of a convalescent COVID-19 patient who had been infected by SARS-CoV-2 D614G assumed from the onset period (July 2020) and then received three doses of an mRNA vaccination (Table S1) via the procedure described in our earlier study (7). The neutralizing activity of MO11 against SARS-CoV-2 variants was quantitatively evaluated by a plaque reduction assay using the authentic virus of major SARS-CoV-2 variants: a variant harboring spike D614G mutation (hereafter, D614G), Delta, Omicron BA.1, BA.2, BA.2.75, BA.5, BQ.1.1, XBB.1, XBB.1.5, XBB.1.16, and EG.5.1. We observed that MO11 neutralized all of the variants tested including the latest (EG.5.1) in a concentration-dependent manner (Fig. 2A and B). The IC_50_ range was 103–883 ng/mL (Fig. 2A and C), showing a potency compared to the RBD-targeting antibody MO1 with IC_50_ 15.67 ng/mL for BA.5 reported in our previous study (7). Of note, complete inhibition was observed at the 1–10 µg/mL range of MO11 (Fig. 2A).

### MO11 binds to the spike by recognizing an epitope outside the RBD

**Fig 3:**
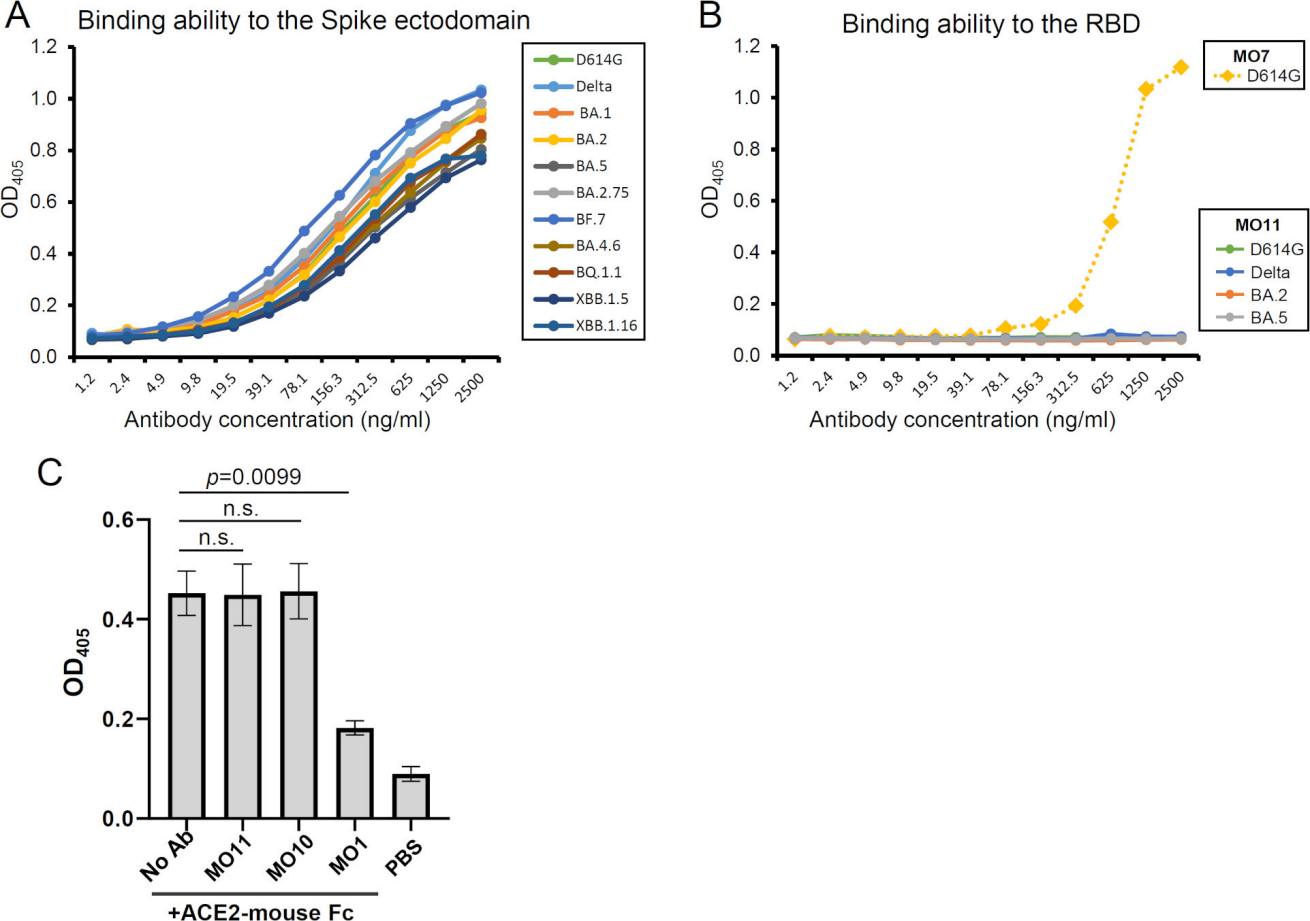
MO11 recognizes an epitope outside of the RBD and does not inhibit the ACE2-spike interaction. (**A**) The binding ability of MO11 to the pre-fusion stabilized spike ectodomain. (**B**) The binding ability of MO11 to the spike RBD. The RBD-targeting antibody MO7 isolated in our previous study (7) was also included as a control. (**C**) A competition enzyme-linked immunosorbent assay was performed to analyze MO11’s ability to inhibit the ACE2-spike interaction. The mean of three independent experiments with the standard error is shown for each group. The data were analyzed by Dunnett’s multiple comparisons test against the no-antibody (No Ab) group. Probability (p)-values < 0.05 were considered significant.

The spike recognition ability was confirmed by an enzyme-linked immunosorbent assay (ELISA) using the pre-fusion stabilized spike ectodomain with the artificial proline mutations (16) and the mutations of SARS-CoV-2 variants. Reflecting the broad neutralizing activity of MO11, spike ectodomains of these variants were recognized by MO11, with no large difference in the results (Fig. 3A). We also tested MO11’s recognition of the spike RBD of several variants: D614G, Delta, BA.2, and BA5. We observed that MO11 did not bind to the spike RBD of all of the variants tested (Fig. 3B), in contrast to the RBD-binding antibody MO7 isolated in our earlier work (7), indicating that the recognition site of MO11 is outside of the RBD.

We next performed a competition evaluation between MO11 and human ACE2 for their ability to bind to the spike ectodomain of BA.2 variant by conducting an ELISA. The detected amount of ACE2 ectodomain fused with mouse Fc was not changed by using MO11 as the competitor (Fig. 3C). The same result was observed when a non-neutralizing antibody targeting outside of RBD isolated in our previous study (7), i.e., MO10, was used as the non-competitive control, because it is reasonable that MO10 does not inhibit the ACE2 binding. In contrast, a neutralizing antibody, MO1, which inhibits ACE2 binding (7), significantly decreased the bound ACE2 amount in this assay. A biolayer-interferometer assay also showed that MO11 did not inhibit the ACE2 binding to spike (Fig. S1). These results indicated that MO11 binds outside of the RBD and has little, if any, influence on the spike-ACE2 interaction.

### MO11 binds to spike subdomain 1 by targeting the epitope exposed in the pre-fusion trimer

**Fig 4:**
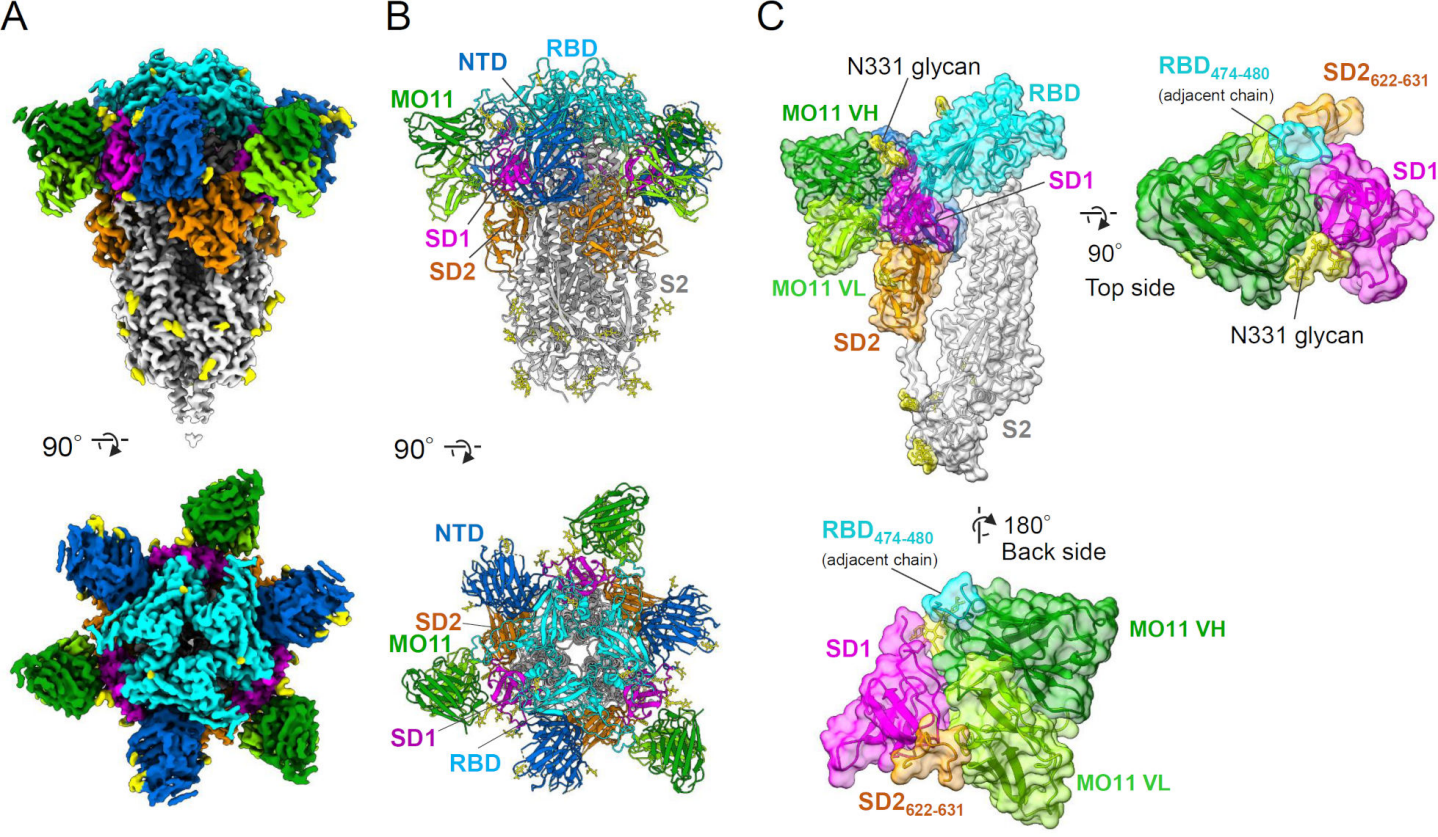
The complex structure of MO11-spike revealed by cryoEM. (**A and B**) The density map (**A**) and the molecular models (**B**) of MO11’s VH/VL – BQ.1.1 spike complex are shown from two different views. Blue: NTD, cyan: RBD, magenta: SD1, orange: SD2, white: S2, yellow: glycans, green: MO11 VH, light green: MO11 VL. (**C**) Close-up views of the MO11-SD1 contact are shown as ribbon models with solvent-accessible surfaces.

CryoEM was applied to elucidate the molecular interaction between MO11 and the BQ.1.1 spike ectodomain. The Fab domain of MO11 and the BQ1.1 spike ectodomain were mixed and purified as a complex in size-exclusion chromatography and used for the cryoEM single particle analysis (Fig. S2). The resulting EM map showed a single conformation between protomers of the trimeric spike-Fab complex. At the beginning of the single particle analysis, low-resolution EM map indicated the presence of C3 symmetry, and thus, a symmetry restraint was applied during the image analysis. The refined EM map was evaluated to be at the global resolution of 2.3 Å (Fig. 4A; Fig. S3 and S4), and the atomic model was built as the complex consists of a spike trimer and three variable heavy and variable light (VH/VL) chains of MO11 (Fig. 4B). The summary of the cryoEM data collection parameters and conditions used for cryoEM data analysis are listed in Fig. S3 and S4.

The EM map and corresponding atomic model of the MO11-BQ.1.1 spike complex are shown in Fig. 4. All three of the RBDs revealed to take a “down” conformation in this complex. Three VH/VLs of MO11 bind to SD1 and occupy the spaces between two adjacent NTDs (Fig. 4B and C). Each SD1 broadly contacts MO11 across its interface of VH and VL domains, and one glycan extending from the N331 of spike SD1 runs along the outer rim of the MO11 VH domain (Fig. 4C). The footprint of MO11 on SD1 does not include the reported mutation sites in SD1, i.e., A570D for B.1.1.7 (Alpha), T547K reported in Omicron BA.1 and BA.1.1, and D574V for some of Omicron BA.2.75 (Fig. 1A; Fig. S5). MO11 also has additional contacts with SD2 and the RBD of an adjacent monomer (Fig. 4C), although the EM density map of those regions of the spike is slightly ambiguous. MO11 has a contact site with a buried surface area that is calculated as 1,983 Å^2^.

### Details of the MO11-SD1 interaction

**Fig 5:**
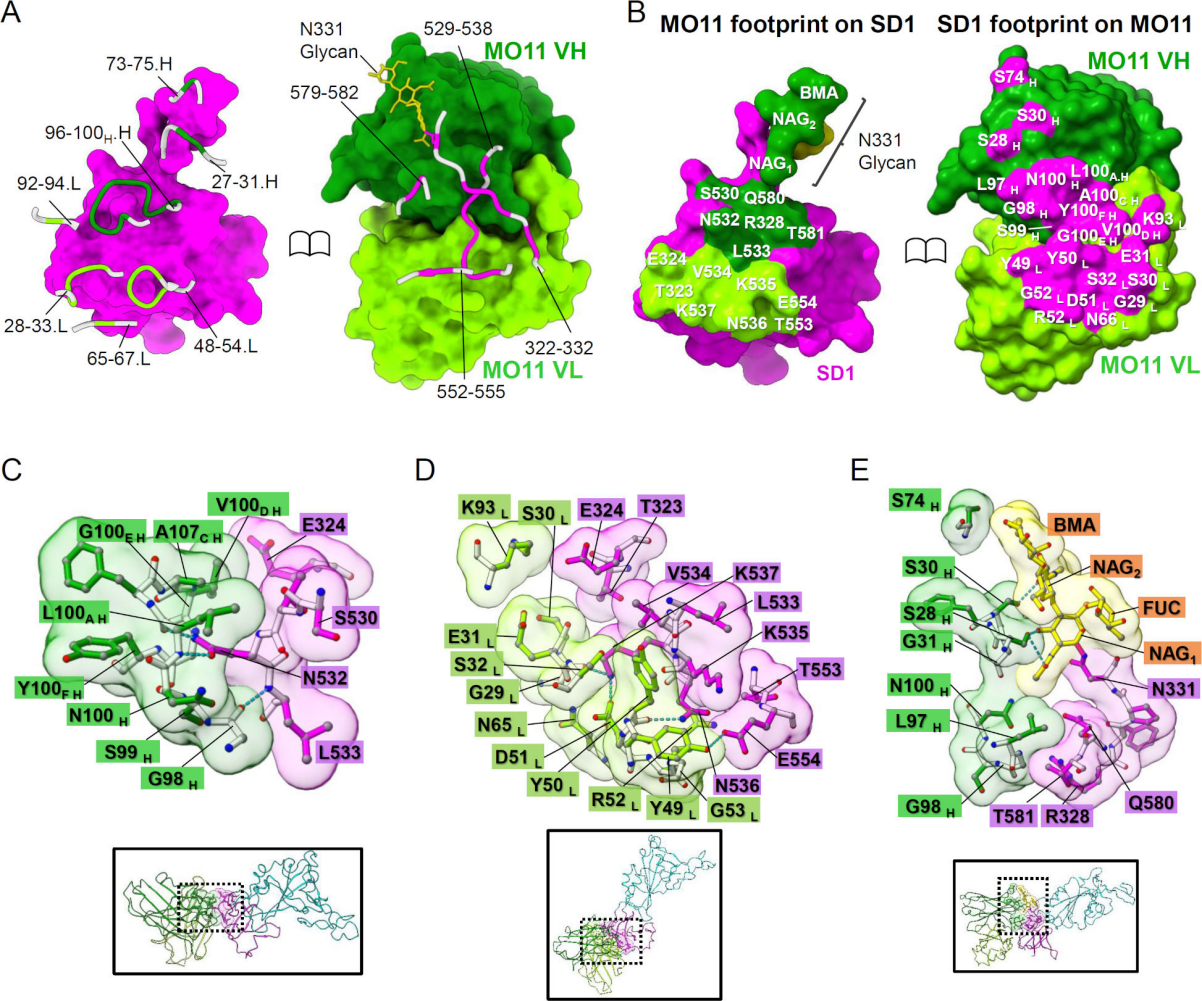
Details of the molecular interaction between MO11 and the spike. (**A**) Close-up views of the MO11-SD1 contact are shown as ribbon models with solvent-accessible surfaces. (**B**) The MO11 VH/VL footprint on the spike SD1 (*top*) and that of spike SD1 on MO11 (*bottom*). (**C**) The interaction around the spike N532. (**D**) The broad interaction between MO11 VL and the spike SD1. (**E**) The interaction around N331 glycan.

The detailed interaction between the MO11 VH/VL and the spike is illustrated in Fig. 5 and summarized in Table S2. MO11 has contacts with several linearly distant regions of SD1; mainly 529–538 as well as 552–555, 322–332, and 579–582. The loop 529–538 is located at the center of MO11’s footprint (Fig. 5A and B) and is in wide contact with MO11’s complementarity-determining regions (CDRs), i.e., H3, L1, and L2. Notably, the CDR H3 loop extending to SD1 surrounds the sidechain of N532 protruding from a flat surface of SD1 (Fig. 5A and C). The amide group of N532 forms bifurcate hydrogen bonds with the carbonyl oxygen and amino nitrogen of MO11’s L100_A H_. Another hydrogen bond between mainchains of spike L533 and CDR H3 G98_H_ is observed nearby.

The following part, spike 534–537, faces the CDRs L2 and L1 (Fig. 5D). The ring of Y50_L_ lays on the SD1 surface around the sidechains of V534 and K535, and the sidechain of N536 forms a hydrogen bond with the Y50_L_ mainchain carbonyl. The amide Nz at the tip of the K537 sidechain is surrounded by the mainchain carbonyl of CDR L1 G29_L_, forming a hydrogen bond, and the sidechain CDR L2 D51_L_ forming a salt bridge. A sidechain of E554, which is spatially located near K535, forms hydrogen bonds with the OH of CDR L2 Y49.

A glycan chain attached to the N331 sidechain is visible in the cryoEM density map and runs along MO11’s VH (Fig. 4 and 5E). The N-acetyl glucosamine (NAG) at N331 (NAG_1_) is extended by a β1–4 NAG (NAG_2_) and an α1–6 fucose (FUC), and the NAG_2_ is further linked to β-D-mannopyranose (BMA). The two NAGs contact the CDR H1 around S28_H_ and S30_H_, and the BMA reaches S74 at the non-CDR loop. The acetyl group of the NAG_1_ contacts the CDRs H1 G31_H_ and Y32_H_ as well as the CDRs H3 L97_H_ and N100_H_. The sidechains of R328, Q580, and T581 positioned nearby also contact the CDRs H3 L97_H_ and G98_H_.

## DISCUSSION

The human mAb MO11 isolated from memory B cells of a patient who had been infected by SARS-CoV-2 and received three doses of an mRNA vaccination demonstrated its broad and effective neutralizing activity against SARS-CoV-2 variants including the recent EG.5.1 (Fig. 2A), reflecting the detectable neutralizing titer of the sera (Table S1). Since the donor was immunized via infection by the D614G strain and considering the onset period and the conventional mRNA vaccine used, it is reasonable to speculate that the obtained MO11 is the antibody targeting the conserved epitope in the SD1 shared among major SARS-CoV-2 variants reported to date. The present study’s mAb screening scale of antibody isolation is small compared to those of other studies, and we did not use any dedicated methodology other than the use of pre-fusion BA.5 spike to sort B cells. The isolation of antibody with cross-reactive activity thus implies the contribution of MO11-like antibodies behind the cross-reactive human sera.

Neutralizing antibodies targeting the SARS-CoV-2 spike’s SD1 have rarely been reported, in contrast to the numerous published descriptions of neutralizing antibodies targeting the RBD or NTD (13). Several research groups have reported SD1-targeting antibodies (Fig. S6); the SD1-targeting antibodies were isolated from individuals with various immune backgrounds (8, 9, 11) or in artificial efforts such as mRNA isolation (12) or a mouse immunization experiment (10). Although all of these SD1-targeting antibodies including MO11 display cross-neutralizing activity due to the conserved nature of SD1, there are several differences among the antibodies in the binding mode and the neutralizing activity. The neutralizing activities of anti-SD1 antibodies are broad but modest compared to the anti-RBD or anti-NTD antibodies, and sometimes unexplained characteristics have been observed due to the unknown neutralizing mechanisms of SD1-targeting antibodies, without inhibition of the ACE2 engagement. For example, Tanaka et al. reported that their SD1 antibody N-612–004 showed partial neutralization activity, reaching a plateau of around 40% neutralization (12). In addition, a fivefold difference in IC_50_ values was reported between the live virus assay and the pseudovirus assay systems in the mouse antibody S3H3 (10). MO11 displayed neutralization activity against authentic live viruses of SARS-CoV-2 variants, and nearly 90% inhibition at the concentration 2.5 µg/mL was observed (Fig. 2A). Interestingly, the epitope residues of the MO11 are fully conserved as shown in the cryoEM structure (Fig. 1B; Fig. S5A), but the IC_50_ values for the variants range from 110.3 ng/mL (XBB.1.16) to 812.2 ng/mL (Delta). This may reflect the difference in the spike characteristics among SARS-CoV-2 variants, such as the dynamics of spike molecules.

MO11 is the first human-derived SD1-targeting neutralizing antibody that stably binds to the pre-fusion spike trimer. There had been no isolation of such antibodies from human sources to date, as all of the reported human SD1-targeting antibodies recognize epitopes hidden in the pre-fusion trimer (11, 12) or contact an NTD (8), resulting in a spike protomer-antibody complex in the cryoEM analyses. The antibody C68.59 reported by Guenthoer et al. was not structurally analyzed in detail, probably because of the spike destabilizing function (9). Recognition of an SD1 epitope on the pre-fusion trimer has been revealed only for the murine antibody S3H3 (10). The exposed surface area of SD1 in the form of a pre-fusion spike trimer is rather small, and thus, the available epitopes are limited. In addition, an adjacent NTD may spatially restrict the route of access to the SD1 as observed for the sd1.040 (8). MO11 and S3H3 have footprints that partially overlap with each other and place the VH/VL at the spaces between the two adjacent NTDs, although their recognition modes are largely different in the epitope, access angle, and usage of the CDRs (Fig. S6C), at least partially reflecting gene differences between human and murine antibodies.

MO11 extensively contacts the glycan chain at the N331 position (Fig. 5A and E) as a unique feature different from the other SD1-targeting antibodies reported to date (Fig. S6B). Because MO11 retained its binding ability to BQ.1.1 spike with a T333A mutation which breaks the N-glycosylation motif at the spike _331_NIT_333_ in an indirect immunofluorescent assay (Fig. S7), the contact between MO11 and the glycan at N331 is dispensable for the interaction. Unlike MO11, the other reported SD1-targeting antibodies do not interact with the N331 glycan by binding distant epitopes on SD1 (Fig. S6B). The large glycan chain extending from N331 may further restrict the available surface area on SD1. Considering the possibility that antibodies’ binding to SD1 may have neutralization activity, we speculate that the glycan may serve as a protecting shield of the spike antigen to limit the availability of the neutralizing epitopes. Indeed, Seow et al. showed that the glycan at N331 disturbs the antibody function, and cleavage by an enzyme enhanced the neutralizing effect of the antibody P008_60 (11). Moreover, Li et al. demonstrated that a glycosylation mutant N331Q showed increased sensitivity to convalescent sera (15). MO11 is not blocked by the glycan and does not remain at a distance from it; rather, it crawls into the hidden epitope behind the glycan, demonstrating the availability of the neutralizing epitope for human immunity.

The neutralizing mechanism of an SD1-targeting antibody is unclear since there are only a few reports of SD1-targeting antibodies, and the role of SD1 in spikes’ function is unknown. MO11 did not completely interfere with ACE2 binding (Fig. 3C; Fig. S1). Although the cryoEM structure of the MO11-spike complex was revealed as a closed form with all down RBD hiding the receptor binding site, recent reports indicated that the spikes of BA.2 and the descendant have an intrinsic preference for all down conformation in the cryoEM analysis (17–19) without lacking the ACE2 binding ability. As the MO11-spike structure showed no obvious structural features that confine the closed form of the spike, it is possible that the temporal up RBD conformation is trapped by ACE2. The lack of inhibition activity against the ACE2-spike interaction is also true of the other SD1-targeting antibodies (8, 11), and thus, the other steps of spike function are probably inhibited. Seow et al. and Guenthoer et al. suggested the destabilization of the spike trimer upon antibody binding as a possible mechanism of their antibodies (9, 11). This is not applicable to MO11 because the binding mode of MO11 revealed by its high-resolution cryoEM structure indicates no conflict upon binding to the spike. Bianchini et al. suggested the stabilization of the spike since, in their simulation, their antibody bound to SD1 and contacted the NTD (8). A stabilization mechanism was also suggested for the murine antibody S3H3 because of the interaction with multi-segments of the SD1 of the spike trimer (10), although the details of the stabilization mechanism are not established. Since MO11 also holds the SD1 across the distant segments of SD1 (Fig. 5A ; Fig. S5A), i.e., the SD1-N and SD1-C labeled in Fig. 1, those segments may pin down the SD1 from the trimer surface, contributing to the stabilization of the SD1 fold and retaining the S1–S2 connection in the context of the dynamic nature of the spike trimer (5, 20), and thereby, MO11 may prevent conversion from pre- to post-fusion conformations.

In summary, our identification of MO11 unveiled the conserved neutralizing epitope on the SD1 of the spike as an actual target of human immunity. A live virus-neutralizing assay of MO11 against broad SARS-CoV-2 variants demonstrated the importance of the SD1 in the activity of the complicated spike functions. Although the neutralizing mechanisms of MO11 and other SD1-targeting antibodies remain to be elucidated, it is speculated that bound antibody restricts or corrupts the conformational changes necessary for spikes to elicit membrane fusion. The availability of the high-resolution structure obtained in this study will provide clues regarding how the spike dynamics are controlled by SD1.

## MATERIALS AND METHODS

### Collection of human blood samples

Blood samples were collected from a patient with COVID-19 who was being followed up at Hyogo Prefectural Kakogawa Medical Center (Kakogawa, Japan) under an informed consent agreement. The patient had received three doses of an mRNA COVID-19 vaccine after contracting a COVID-19 infection in July 2020. Sera and peripheral blood mononuclear cells from the patient were prepared as described (7).

### Isolation of human antibodies’ genes from the donor’s peripheral blood mononuclear cells

The isolation of human antibody genes was performed using Ecobody technology (21) (iBody Inc., Nagoya, Japan) as described (7). Briefly, the recombinant SARS-CoV-2 pre-fusion stabilized spike of the BA.5 variant was fluorescently labeled, and memory B cells were screened as single cells by a FACS SH800S system (Sony, Tokyo). The genes of each antibody variable region were amplified by PCR from the cDNA of each cell. Candidates were screened by an ELISA for the Fabs produced by *Escherichia coli* cell-free protein synthesis (PUREfrex 2.0, GeneFrontier, Kashiwa, Japan) using plasmids containing VH and VL genes from each cell, according to established protocols (21).

The sequence of the MO11 VH gene was subcloned into the pFUSEss-CHIg-hG1 plasmid (InvivoGen, San Diego, CA). The VL gene was first combined with the Cλ sequence derived from the plasmid pFUSEss-CLIg-hL2 (InvivoGen) by overlap PCR and then inserted into the pCAGGS plasmid (22) which was modified to contain an IL2 signal sequence. The sequence was confirmed by a capillary electrophoresis sequencer (model DS3000, Hitachi High-Tech, Tokyo) to confirm the sequences.

### Antibody expression and purification

Antibody expression and purification were performed as described (7). Briefly, MO11 was expressed by the co-transfection of the antibody plasmids described above using the Expi293F Expression System (Thermo Fisher Scientific, Waltham, MA), and the antibody was purified by affinity purification with rProtein A Sepharose (Cytiva, Marlborough, MA).

### Preparation of the pre-fusion spike, RBD, and human ACE2 proteins

Proteins of the pre-fusion stabilized spike ectodomain of each SARS-CoV-2 variant, RBD (spike 334–528), and the ectodomain of human ACE2 (1-614) with mouse Fc were prepared as described (7, 23). Briefly, the pCAGGS plasmid harboring each gene was transfected to HEK293T cells by polyethyleneimine, and the supernatant was collected 4–5 days later. The Expi293F expression System (Thermo Fisher Scientific) was also used according to the manufacturer’s protocol. As all of the gene constructs included C-terminal His_6_ sequences, the expressed proteins were purified by nickel-charged nitriloacetic acid agarose (Qiagen, Hilden, Germany).

### Evaluation of purified proteins

An Amicon Ultra centrifugal filter (Sigma Chemicals, St. Louis, MO) was used to concentrate the purified proteins and to replace the solvent. The concentration of each purified protein was evaluated by measuring the absorbance at the 280 nm wavelength by a NanoDrop spectrophotometer (Thermo Fisher Scientific), and the purity was confirmed by an SDS-PAGE assay.

### Enzyme-linked immunosorbent assay

The ELISA was performed as described (7). Briefly, 96-well ELISA plates (Corning, New York, NY) were coated by the pre-fusion stabilized spike ectodomain or the RBD (at 100 ng/well) in carbonate-bicarbonate buffer at 4°C overnight. The next day, each well was washed twice with phosphate-buffered saline (PBS)−0.1% Tween 20 (PBST) and incubated overnight at 4°C with blocking buffer (1% bovine serum albumin with PBS). Each antibody at the indicated concentration was added as the first antibody and incubated for 1 hour at 37°C. After the plate was washed with a wash buffer, horseradish peroxidase (HRP)-conjugated goat anti-human IgG (1:10,000 dilution, Abcam, Cambridge, MA) was added as the secondary antibody for 1 hour at 37°C. After incubation with ABTS solution (Roche Diagnostics, Indianapolis, IN) for 40 minutes and after the reaction was stopped by the addition of 1.5% (wt/vol) oxalic acid dehydrate solution, the optical density at 405 nm (OD_405_) was measured by a microplate photometer (Multiskan FC, Thermo Fisher Scientific).

For the competition assay, ACE2-mouse Fc was mixed with each antibody or the buffer at the indicated concentration and added as the first antibody. The subsequent steps were the same as those described above, with the exception that HRP-conjugated anti-mouse IgG (1:10,000 dilution, Abcam) was used to detect the ACE2-mouse Fc in parallel with the use of the HRP-conjugated anti-human IgG.

### Viruses

The SARS-CoV-2 strain harboring the D614G mutation was provided by BIKEN Innovative Vaccine Research Alliance Laboratories [Osaka, Japan; DNA Data Bank of Japan (DDBJ): accession no. LC644163]. The following SARS-CoV-2 variants were provided by Japan’s National Institute of Infectious Disease (Tokyo): the Pango lineage AY.122 (Delta, EPI_ISL_2158617), BA.1.18 (Omicron BA.1, EPI_ISL_7418017), BA.2 (EPI_ISL_9595859), BA.2.75 (EPI_ISL_13969765), BA.5 (EPI_13241867), BQ.1.1 (EPI_ISL_15579783), XBB.1 (EPI_ISL_15669344), XBB.1.5.19 (XBB.1.5 EPI_ISL_ 16889601), and XBB.1.16 (EPI_ISL_17718585). EG.5.1 (Hyo-23806755) was received from Hyogo Prefectural Institute of Public Health Science. The viruses were propagated by the infection of Vero E6 (TMPRSS2) cells (24) in 2% fetal bovine serum (FBS) containing Dulbecco’s modified Eagle medium (DMEM) in order to create a stock of each virus.

### Plaque reduction neutralization test

Vero E6/TMPRSS2 cells (2 × 10^5^ cells/well) were seeded on 12-well plates (Corning) and cultured overnight with 5% CO_2_ at 37°C. The plates were washed once with DMEM (without FBS). Each diluted antibody in DMEM (without FBS) was mixed with 100 plaque-forming units of SARS-CoV-2 and incubated for 1 hour at 37°C. The virus–antibody mixture was added to the Vero E6/TMPRSS2 cells for 1 hour with 5% CO_2_ at 37°C. After the inoculum was removed, the infected cells were washed twice with PBS and cultured for 3–6 days at 37°C with 5% CO_2_ in 1.6% methylcellulose containing 2% FBS DMEM. After the supernatants were removed, the cells were washed twice with PBS and fixed with 80% methanol for 1 hour at room temperature. The cells were stained with 1% crystal violet in 50% methanol, and the visualized plaques were counted. The ratio of neutralization was obtained by dividing the number of plaques obtained without the antibody by the number of plaques obtained with the antibody. The plaque reduction neutralization test result for each sample was evaluated using each of the living SARS-CoV-2 variants in a biosafety level 3 laboratory.

### Cryoelectron microscopy

The MO11-BQ.1.1 spike ectodomain complex was prepared as described previously (7). Briefly, the Fab domain of MO11 obtained by papain digestion was mixed with the purified BQ.1.1 spike ectodomain and subjected to the size exclusion column chromatography by a Sephacryl S-300 HR column (Cytiva, Marlborough, MA) equilibrated with 20 mM Tris-HCl pH8.0 150 mM NaCl using the ÄKTA pure system (Cytiva, Marlborough, MA). The sample was concentrated to 8.6 mg/mL by ultrafiltration as described above and the detergent, fluorinated Fos-Choline −8 (anatrace, Maumee, OH) was mixed to the final concentration of 2 mM just before the grid vitrification. Three microliter of the sample was applied to a freshly glow-discharged Quantifoil holey carbon grid (R0.6/1.0, Cu, 300 mesh), using a Vitrobot Mark IV (FEI). The conditions were as follows: temperature, 8°C; blot force, 15; blotting time, 4 s; humidity, 100%. The sample grids were plunge-frozen in liquid ethane and stored until use.

The cryoEM data collection was performed as described previously (7). The detailed information is summarized in Fig. S3. The resolution of the refined EM map was estimated at the Fourier Shell Correlation (FSC) value of 0.143 between two independent half maps (Gold-Standard FSC; Fig. S4D), and the final map is shown and colored with local resolution in Fig. S4D.

The refined EM map was utilized for the atomic model building of the MO11 Fab and BQ.1.1 complex. BA.1 spike structure of our earlier study (PDBID: 8H3N) (7) was used as the starting model, and the amino acid sequence was changed to include mutations of BQ.1.1 by the UCSF ChimeraX 1.6 (25). MO11 Fab model was prepared using the AlphaFold 2.2.0 (26). These initial models were fitted into the cryo-EM map by UCSF ChimeraX and inspected by Coot 0.9.8.1 (27). A density-modified map was calculated by phenix.resolve of PHENIX 1.20.1 (28) and referred during model refinement. The model was iteratively refined by Coot 0.9.8.1, phenix.real_space_refine of PHENIX 1.20.1, and ISOLDE (29) implemented in UCSF ChimeraX 1.6. Glycans were modeled by the carbohydrate module of the Coot 0.9.8.1. The final model was subjected to the validation by phenix.validation of PHENIX1.20.1, and glycan structures were assessed by Privateer (30) implemented in CCP-EM 1.6.0 (31). The software ChimeraX was used for making illustrations of molecular structures.

## Data Availability

The cryo-EM map and coordinates of MO11-spike BQ.1.1 complex have been deposited at the Electron Microscopy Data Bank and Protein Data Bank as EMD-38372 and PDB ID 8XI6, respectively.

## SUPPLEMENTAL MATERIAL

Supplemental material jvi.00416-24-s0001.pdf Figures S1 to S7; Tables S1 and S2.

ASM does not own the copyrights to Supplemental Material that may be linked to, or accessed through, an article. The authors have granted ASM a non-exclusive, world-wide license to publish the Supplemental Material files. Please contact the corresponding author directly for reuse.
