# Supplementary material for: Epitopes of an antibody that neutralizes a wide range of SARS-CoV-2 variants in a conserved subdomain 1 of the spike protein
Source: J Virol. 2024 Apr 16;98(5):e00416-24. doi: 10.1128/jvi.00416-24 (PMC11092320; doi:10.1128/jvi.00416-24)
Supplement: Supplemental material — Figures S1 to S7; Tables S1 and S2. [file jvi.00416-24-s0001.pdf]

Figure S1

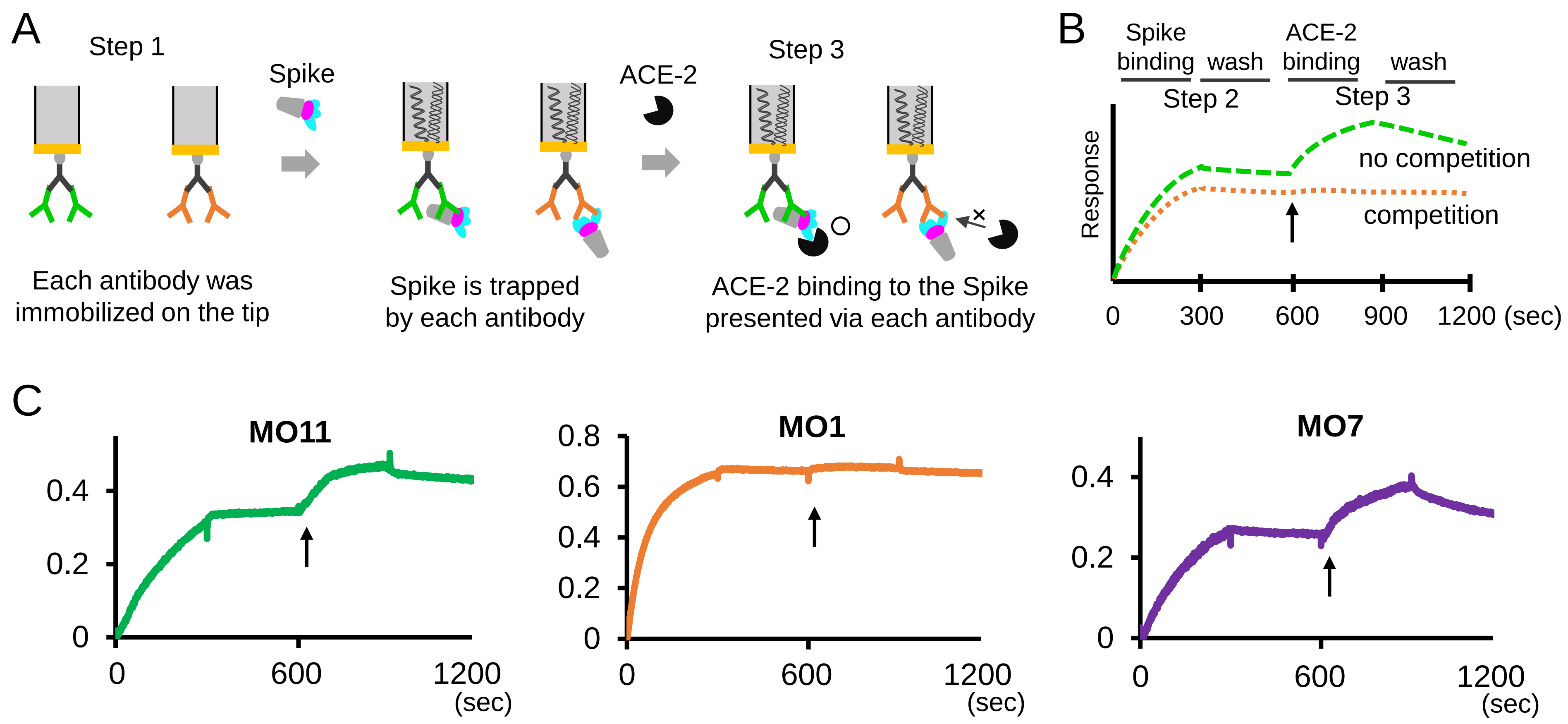

Figure S1. Biolayer interferometer (BLI) assay to test the competition between the ACE2 and the MO11.
(A) A schematic illustration of the BLI-based competition assay. First, each antibody is immobilized on the sensor tip via anti-Ig antibodies (Step 1). The tip was soaked in the Spike BA.2 solution, thereby Spike proteins were trapped on the tip (Step 2). After washing the sensor tip, it was soaked into 100 nM human ACE-2 solution to detect the ACE2 binding to the Spike presented via each antibody (Step 3). **(B)** An illustration of assumed BLI chart. If the antibody-Spike interaction competes with the ACE2 binding, the BLI response is expected to be suppressed. **(C)** Representative experimental data for the antibody vs ACE2 competition assay. The loaded antibodies were indicated on each chart. Allows indicated the timing of the ACE2 loading. It was suggested that ACE2 could bind to the spike without competing with the MO11 (left). MO1 (middle) and MO7 (right) were also tested for comparison since our previous research has revealed that MO1 inhibits ACE-2-spike RBD interaction while MO7 does not (Ishimaru et al. 2022).

Table S1

Table S1. Background of blood donor and the neutralization titers of the serum.

	Age	Sex	Infection* (onset)	Severity	Number of vaccinations	Last vaccination (months from collection)	Blood collection (months from onset)	Neutralizing antibody titers†			
								D614G	BA.5	BQ.1.1	XBB
Donor	45	female	Jul, 2020	mild	3	Feb, 2022 (2)	Apr, 2022 (21)	512	128	4	4

*The D614G is assumed to be the infected strain considering the prevalence in Japan around Jul, 2020.
†The neutralizing antibody titer is defined as described previously[7].

Figure S2

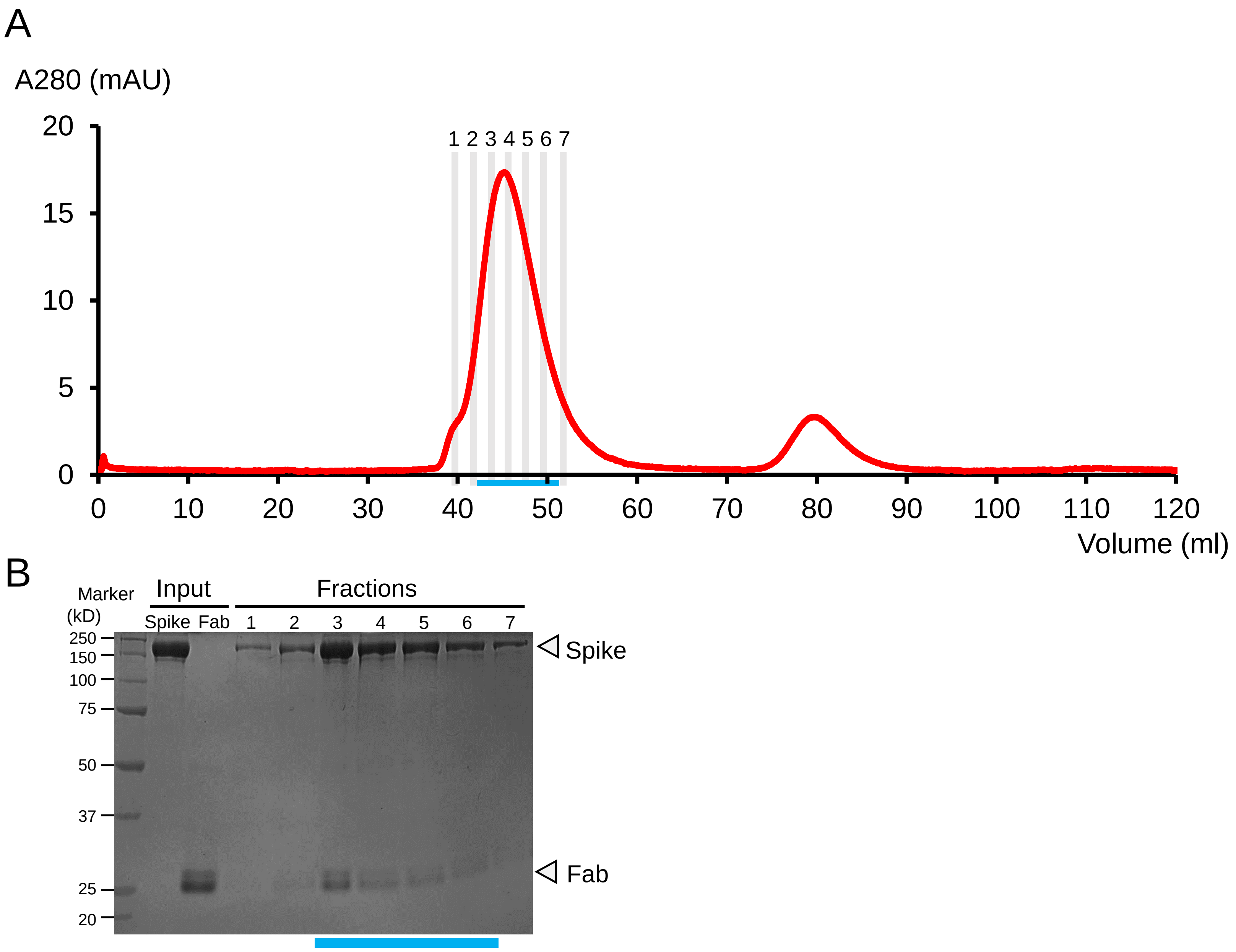

Figure S2. Purification of MO11-spike complex by size exclusion column chromatography.
(A) A chromatography chart of size-exclusion column chromatography. Pre-fusion stabilized Spike ectodomain trimer containing mutations of BQ.1.1 variant and Fab of MO11 were mixed at the ratio of 1:1.5 and injected as input. The fractions subjected to the SDS-PAGE assay are indicated as numbers. **(B)** Confirmation of MO11-spike complex by SDS-PAGE. The gel was staining by CB. The positions of the Spike and Fab were indicated by arrow heads at right. The cyan bars in (A) and (B) indicated the fractions used as the sample for the cryoelectron microscopy.

Figure S3

A

	Information
**Data collection and processing**	
Device and equipment	CRYO ARM 300 (JEOL, Tokyo), SPring-8 EM01CT
	Cold field emission gun
	Omega-type in-column Energy filter
	Gatan K3 camera (Gatan AMETEK, CA USA)
Operation Voltage (300 kV)	300 kV
Nominal Magnification	60,000x
Pixel size (Å)	0.752
Dose rate (e^-/pix/s)	10
Electron exposure e^-/Å^2	50
Defocus range (µm)	-1.2 to -1.6
Image-shift matrix	7x7
Camera mode	Correlated double-sampling (CDS), counting mode
Symmetry applied	C3
Final particle number	246,238
Map resolution (Å)	2.3
FSC threshold	0.143
**Model Refinement**	
PDB, EMDB	8XI6, EMD-38372
Initial Model used (PDB code)	8H3N
Model resolution (Å)	2.35
FSC threshold	0.5
Model composition	
Non-hydrogen atoms	29262
Protein residues	3696
Ligands	NAG:51, FUC:6, BMA:3
B factors (Å^2)	
Protein	64.74
Ligand	83.52
R.m.s deviations	
Bond length (Å)	0.002
Bond angles (°)	0.531
MolProbity score	1.29
Clash score	3.36
Rotamer outliers (%)	1.45
Ramachandran plot (%)	
Favored	97.84
Allowed	2.16
Disallowed	0
CaBLAM outliers(%)	1.19

B

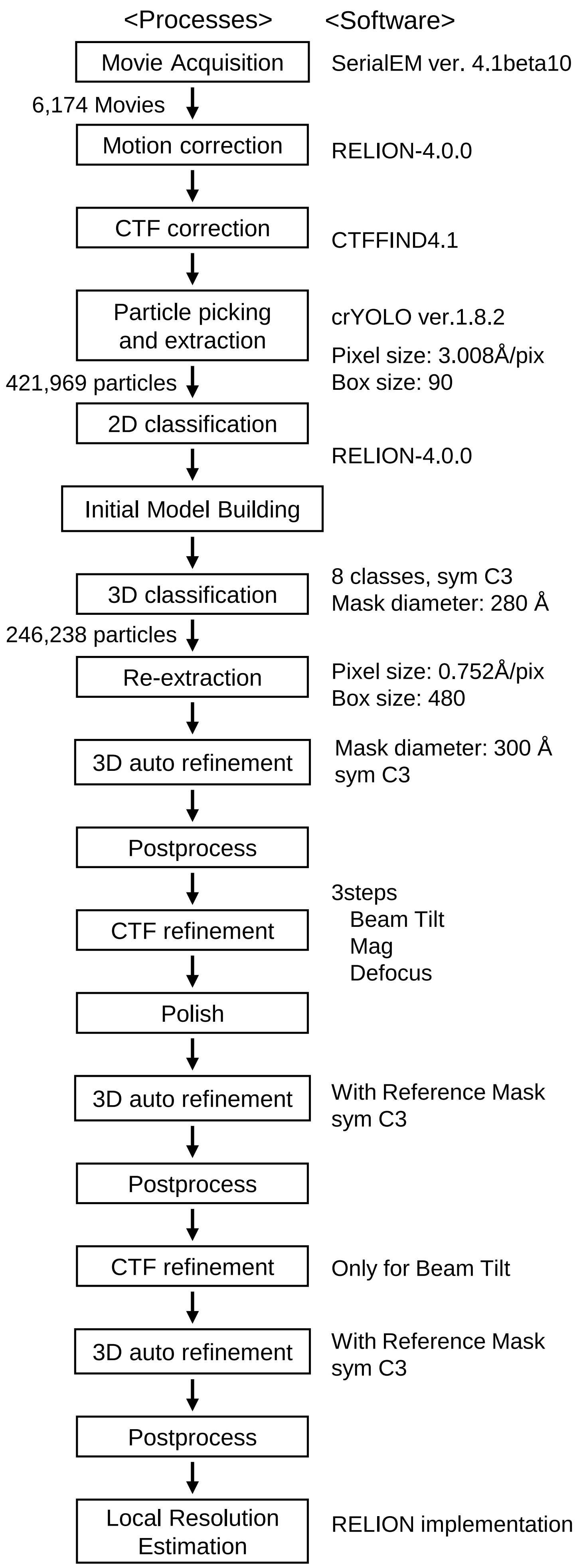

Figure S3. Summary of CryoEM image acquisition and data processing.
(A) Information of image acquisition and data processing. **(B)** Flowchart of CryoEM processing with information of used software. The references of software are as follows; Serial EM ver. 4.1beta (Mastronarde, 2005); CTFFIND4.1 (Rohou and Grigorieff, 2015); RELION-4.0.0 (Kimanius et al., 2021); crYOLO ver. 1.8.2 (Wagner et al., 2019).

Figure S4

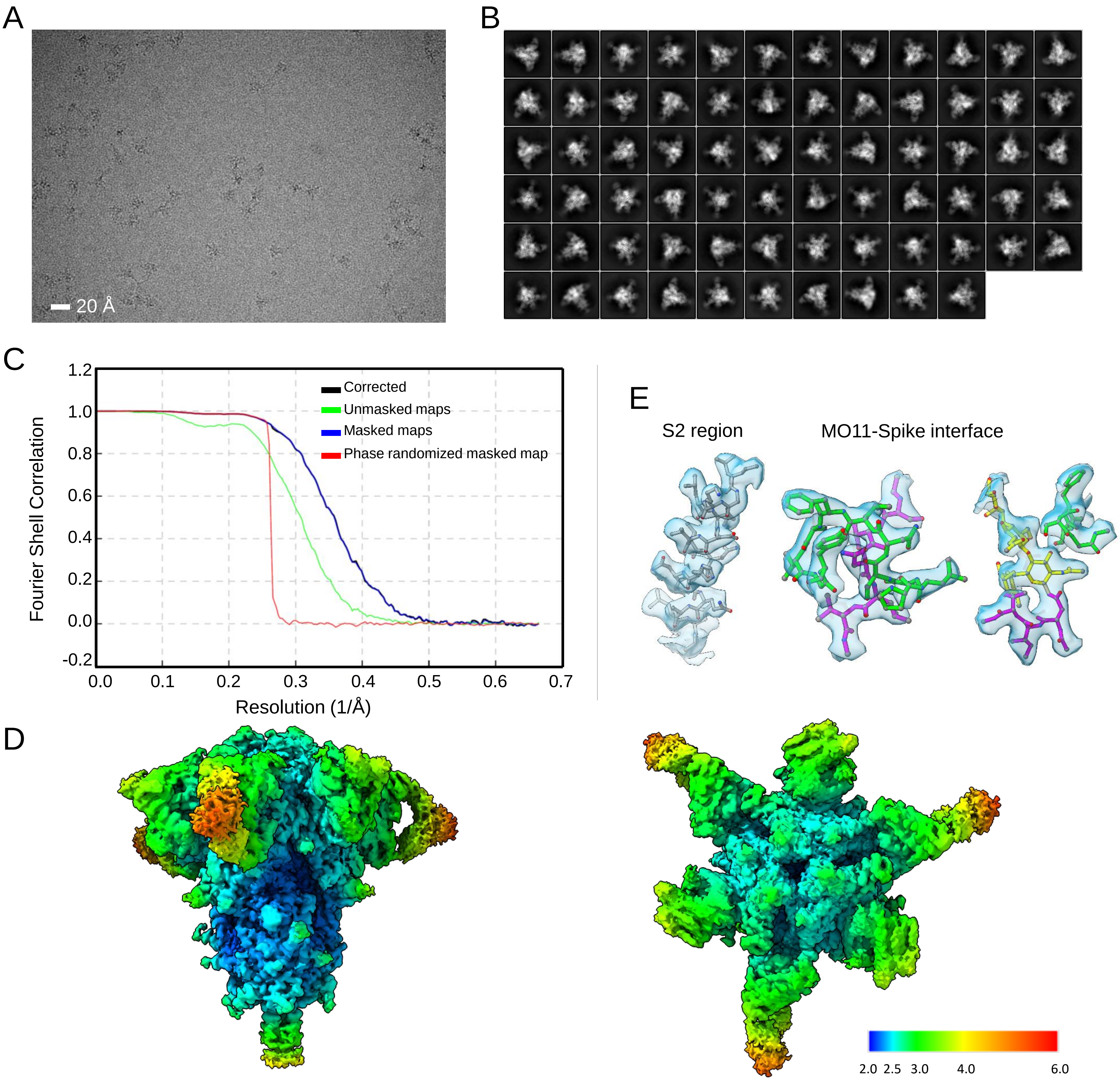

Figure S4. CryoEM image processing of MO11-BQ.1.1 spike complex.
(A) A representative micrograph after motion-correction. **(B)** 2D class averages selected and used for initial model building. **(C)** Gold-standard Fourier shell Correlation curves for the CryoEM map. **(D)** Estimated local resolution distribution for the CryoEM map on the final map. **(E)** The superpositions of EM map and the atomic model at representative regions of MO11-BQ.1.1 spike. The maps and models are shown as transparent surfaces and ball-and-stick models, respectively. For clarity, bonds are colored as follows; gray: S2 region, magenta: SD1 of spike, green: heavy chain of MO11, yellow: glycan on the spike.

Figure S5

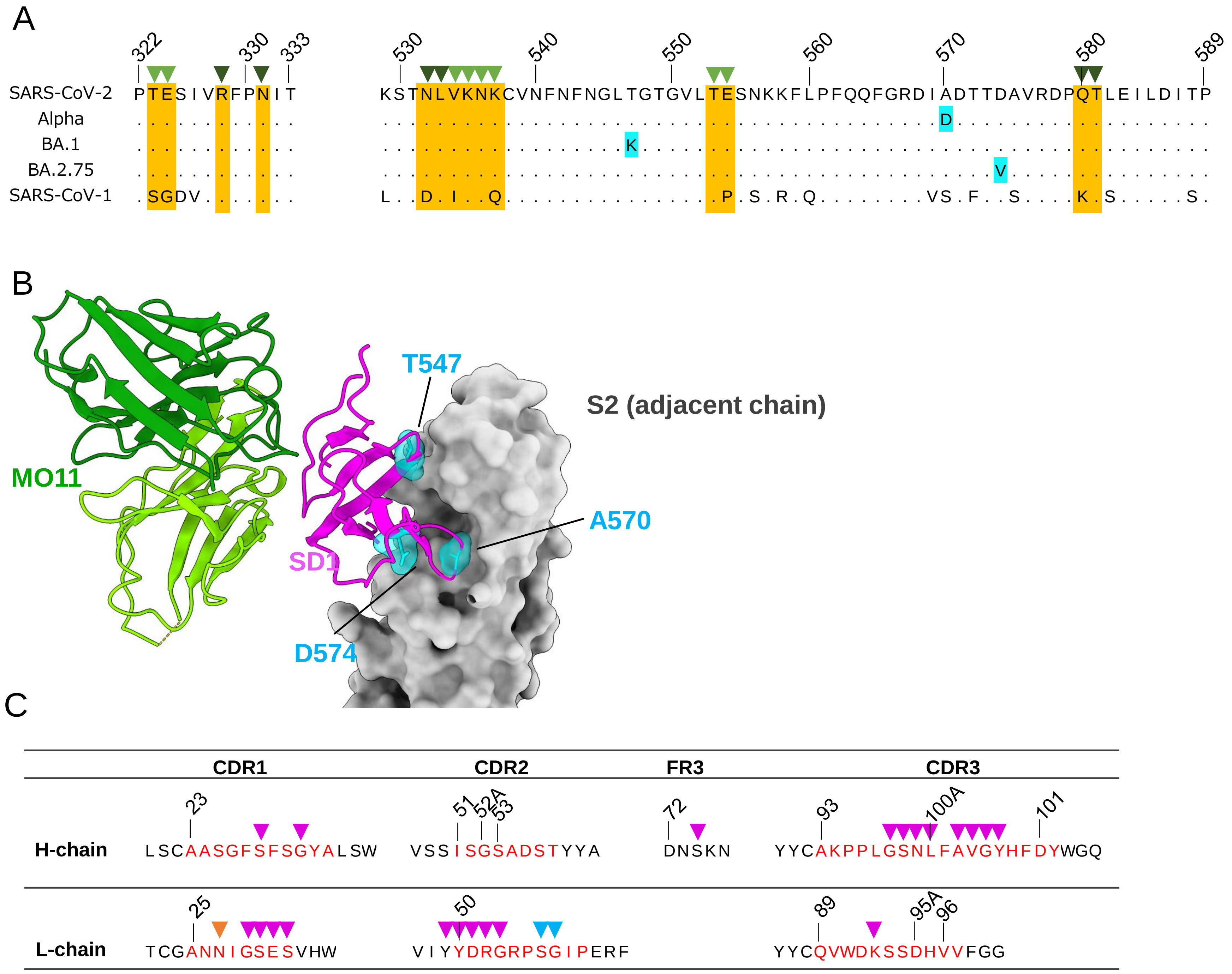

Figure S5. SD1 amino acid sequence and the reported mutations.
(A) Amino acid sequence of the SD1 regions residues, SD1-N: 321-333 and SD1-C: 529-589. The variants with mutation in the SD1, that is, the Alpha (B.1.1.7), BA.1 or BA.1.1, and BA.2.75 were shown. There were no other mutations in major variants. For reference, the sequence of SARS-CoV-1 Spike was also aligned at the bottom. The contact sites of the MO11 were indicated by arrow heads colored by light or dark green for VL or VH, respectively. **(B)** The mutation sites in the SD1 is out of the MO11 footprint. The reported mutation sites T547, D547, and the A570 are located at the opposite side of MO11 binding site which contacts to the S2 of adjacent chain. **(C)** Amino acid sequences of the MO11 H and L chains involved in the interaction. Footprint residues were indicated by arrow head. The color codes are as follows; cyan: RBD, magenta: SD1, and orange: SD2. The residue numbers were also indicated following the Kabat numbering. CDRs of the MO11 were indicated as red letters.

Table S2

Table S2. Summary of interaction between spike and MO11.

Domains	Spike residues	MO11 residues	Possible interaction	Notes	Figure
SD1	T323	S30.L	van der Waals interaction		Figure 4D
	E324	K93.L	Electrostatic interaction		Figure 4D
		$V100_D$.H	van der Waals interaction		Figure 4C
	R328	G98.H	van der Waals interaction		Figure 4E
	N331-NAG1	S28.H	Hydrogen bond (O3-OG)		Figure 4E
		S28.H	Hydrogen bond (O7-OG)		Figure 4E
		N100.H	van der Waals interaction		Figure 4E
	N331-NAG2	S30.H	Hydrogen bond (O7-OG)		Figure 4E
		S28.H	van der Waals interaction		Figure 4E
	N331-BMA	S74.H	Hydrogen bond (O4-O)	Ambiguous density	Figure 4E
	S530	$L100_A$.H	van der Waals interaction		Figure 4C
	N532	$L100_A$.H	Hydrogen bond (ND2-O)		Figure 4C
		$L100_A$.H	Hydrogen bond (OD1-N)		Figure 4C
		G98.H	van der Waals interaction		Figure 4C
		S99.H	van der Waals interaction		Figure 4C
		N100.H	van der Waals interaction		Figure 4C
		$A100_C$.H	van der Waals interaction		Figure 4C
		$G100_E$.H	van der Waals interaction		Figure 4C
		$Y100_F$.H	van der Waals interaction		Figure 4C
	L533	G98.H	Hydrogen bond (N-O)		Figure 4C
		Y50.L	van der Waals interaction		Figure 4D
	V534	S32.L	van der Waals interaction		Figure 4D
		Y50.L	van der Waals interaction		Figure 4D
	K535	Y50.L	van der Waals interaction		Figure 4D
	N536	Y50.L	Hydrogen bond (ND2-O)		Figure 4D
		R52.L	van der Waals interaction		Figure 4D
		G53.L	van der Waals interaction		Figure 4D
	K537	G29.L	Hydrogen bond (NZ-O)		Figure 4D
		D51.L	Salt bridge (NZ-OD2)		Figure 4D
		S30.L	van der Waals interaction		Figure 4D
		E31.L	van der Waals interaction		Figure 4D
		S32.L	van der Waals interaction		Figure 4D
		N66.L	van der Waals interaction		Figure 4D
	T553	R52.L	van der Waals interaction		Figure 4D
	E554	Y49.L	Hydrogen bond (OE2-OH)		Figure 4D
	Q580	L97.H	van der Waals interaction		Figure 4E
	T581	L97.H	van der Waals interaction		Figure 4E
		G98.H	van der Waals interaction		Figure 4E
RBD	N477*	D55.H	Hydrogen bond (ND2-O)	Ambiguous density	
		S56.H	Hydrogen bond (OD1-OG)	Ambiguous density	
SD2	H625	N27.L	van der Waals interaction	Ambiguous density	
	Q628	N27.L	Hydrogen bond (ND2-OD1)	Ambiguous density	
		N27.L	Hydrogen bond (OD1-ND2)	Ambiguous density	

* S477N mutation shared among the Omicron lineage

Figure S6

A

	MO11	S3H3	sdl.040	P008_60	N-61-004	C68.59
Source	Human	Mouse	Human	Human	Human	Human
Citation	This study	Hong et al.	Bianchini et al.	Seow et al.	Tanaka et al.	Guenthoer et al.
PDB ID	8XI6	7WK9, 7WKA	8D48	7ZBU	7S0E	NA
Spike	Spike BQ.1.1 ectodomain	Spike BA.1 Ectodomain	Spike wildtype ectodomain	Spike wildtype ectodomain	Spike wildtype ectodomain	Spike ectodomain
Spike form	Trimer	Trimer	Protomer	Protomer	Protomer	NA
RBD form	All "Down"	1"UP", 2 "Down" or All "Down"	"Down"	"Down"	"Down"	NA
Resolution (Å)	2.3	3.48, 3.64	3.7	4.31	4.9	6.7
N331 glycan	Broad contact	Not modeled	No contact	Partial contact	Not modeled	NA
NTD conflict	No	No	Yes	Yes	Yes	NA
Suggested effect	Trimer stabilization	Trimer stabilization	Trimer stabilization	Trimer destabilization	Trimer destabilization	Trimer destabilization

NA: not available

B

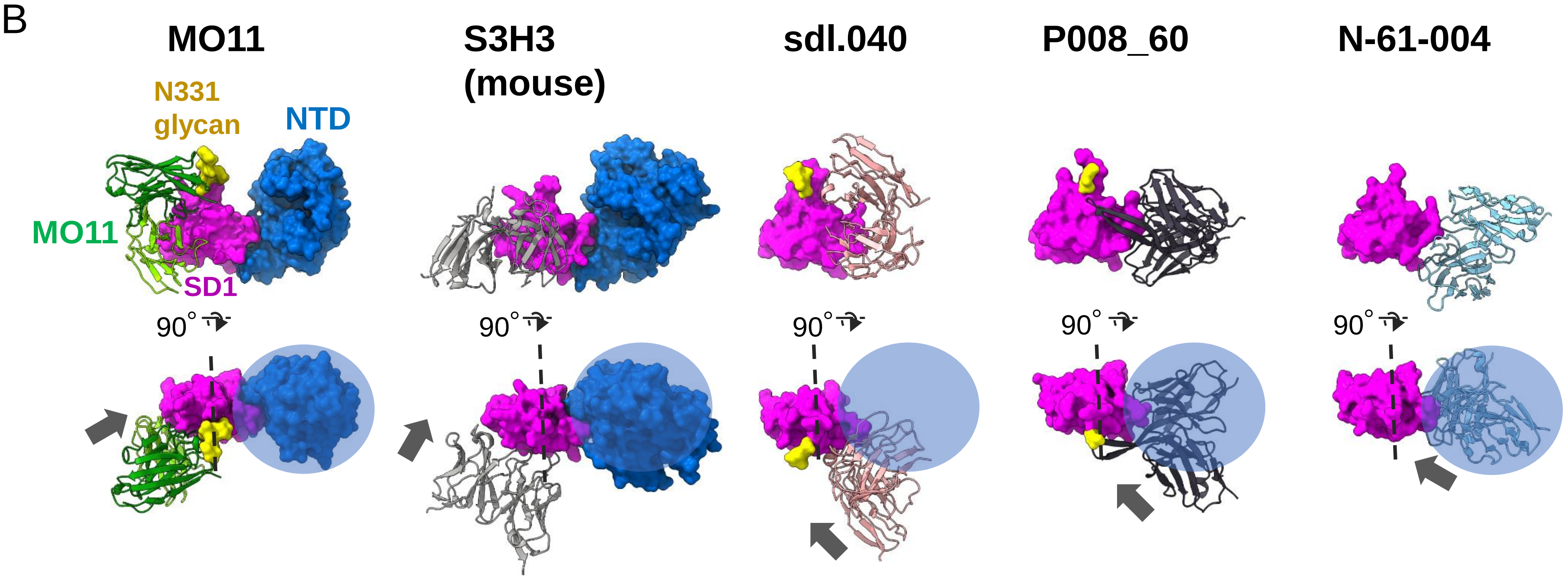

C

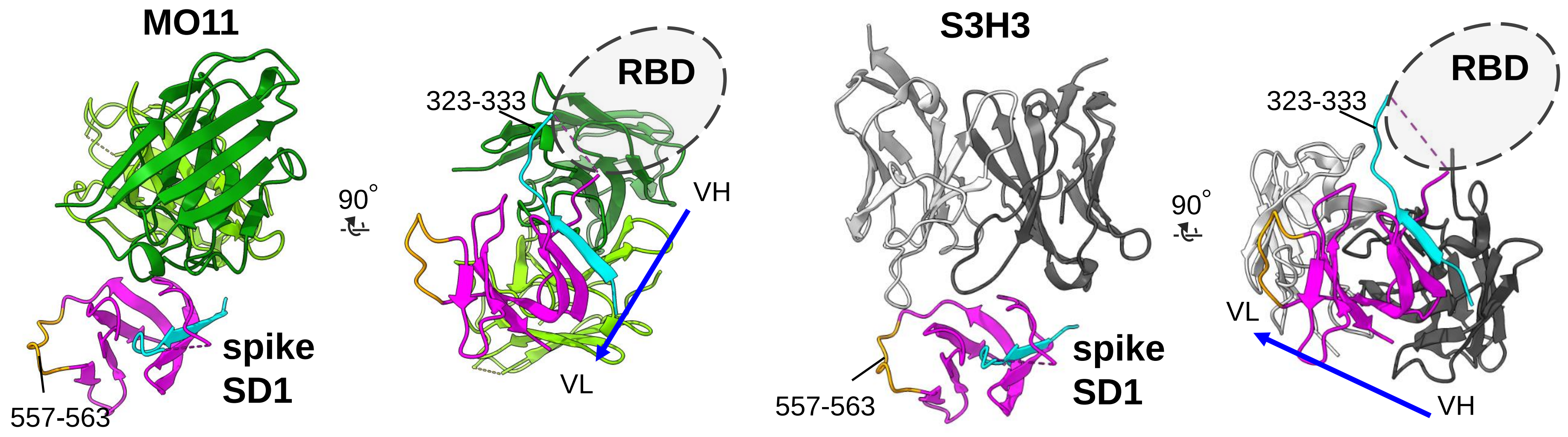

Figure S6. Comparison among SARS-CoV-2 SD1 targeting antibodies.
(A) Summary about the reported SARS-CoV-2 neutralizing antibodies targeting SD1. **(B)** Difference of binding mode for the SD1. MO11 and the murine mAb approaches from the left side in these views, while sdl.40, P008_60 and N-61-004 binds from the right side. Since the right side is near the NTD of adjacent spike protomer, the latter antibodies will conflict with NTD upon binding on the spike trimer. The NTD positions were indicated by transparent blue circles. The N331 glycans are colored by yellow if modeled. MO11 is a unique antibody which contacts to the N331 glycan, in contrast to other SD1-antibodies which avoid the N331 glycan positions. **(C)** Difference between the MO11 and the S3H3. Their VH to VL axes (blue arrows) are different approximately by 90º. As the result, MO11 widely covers the spike 323-333 fragment (cyan), while S3H3 covers it partially. On the other hand, MO11 distant from the spike 557-563 fragment (orange), while the S3H3 reaches that. As the result, they recognize the overlapping epitope in different ways.

Figure S7

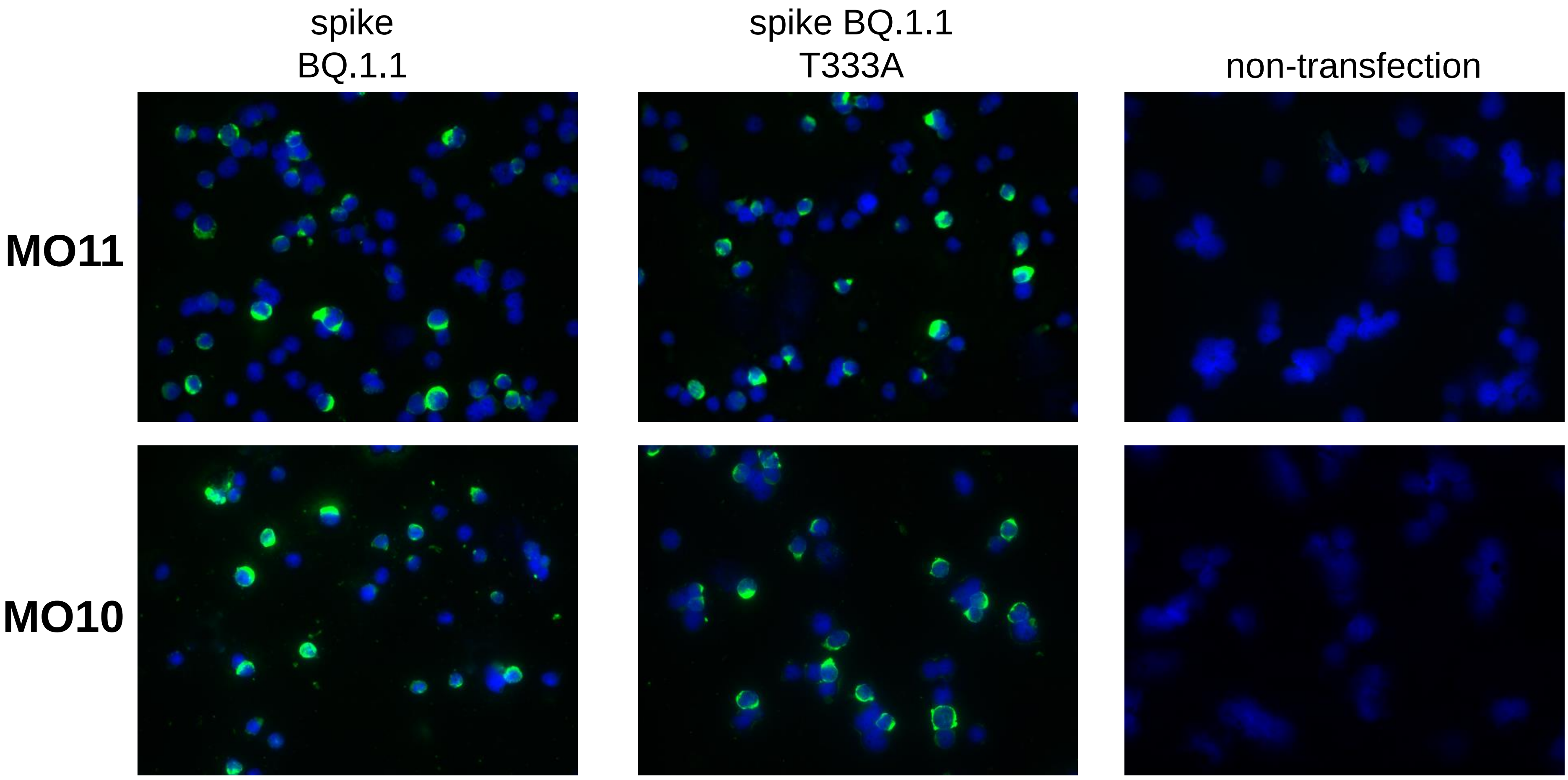

Figure S7. Contribution of the N331 glycan in the molecular interaction between MO11 and spike.
A single point mutation T333A was introduced in BQ.1.1 spike at the N-glycosylation motif $_{331}NIT_{333}$ to break the motif and thereby to exclude the glycan. HEK293T cells were transfected by expression plasmids encoding spike ectodomain of BQ.1.1 or that with the T333A mutation, and then subjected to the indirect immunofluorescent assay. Non-transfected cells were also used for a negative control. Human mAb MO10 (reference 14) which recognizes undetermined epitopes conceivably in S2 was used as a positive control. The fluorescent signals of Alexa 488 conjugated to anti-human IgG were observed for both of spike BQ.1.1 and spike BQ,1,1 T333A expressing cells using MO11 to detect the expressed antigens. Since the N331 glycan is assumed to be lost in the spike BQ.1.1 T333A due to the break of the N-glycosylation motif, this result indicated that the N331 glycan of the spike is dispensable for MO11 to bind to the epitope on SD1.
